# Clinical and economic impact of a specialty care management program among patients with multiple sclerosis: a cohort study

**DOI:** 10.1177/1352458510373487

**Published:** 2010-08

**Authors:** H Tan, J Yu, D Tabby, A Devries, J Singer

**Affiliations:** 1 HealthCore Inc., Wilmington DE, USA.; 2 Department of Neurology, Drexel University, Philadelphia, PA, USA.

**Keywords:** care management, cohort study, healthcare costs, hospitalization rate, medication adherence and persistence, multiple sclerosis, specialty pharmacy

## Abstract

**Background:** To evaluate the clinical and economic impact of a specialty care management program among patients with multiple sclerosis.

**Methods:** This retrospective cohort analysis included patients aged ≥18 years with ≥2 claims of multiple sclerosis diagnosis and ≥1 multiple sclerosis medications from 1 January 2004 to 30 April 2008. The outcome metrics included medication adherence and persistence, multiple sclerosis-related hospitalization, and multiple sclerosis-related cost. Multivariate analyses were performed to adjust for demographics and clinical characteristics.

**Results:** Among the 3993 patients identified, 78.3% participated in the program and 21.7% did not. Over 12 months, medication adherence and persistence improved among participants but deteriorated among non-participants (medication possession ratio change: +0.08 vs −0.03, *p* < 0.001; persistence change: +29.2 days vs −9.2 days, *p* < 0.001). Multiple sclerosis-related hospitalization decreased from 9.6% to 7.1% for participants, whereas it increased from 10.1% to 12.0% for the non-participant group (*p* < 0.001). Multiple sclerosis-related medical spending (non-pharmacy) decreased among participants, but it increased among non-participants (mean: −US$264 vs + US$1536, *p* < 0.001). Total multiple sclerosis-related cost for both groups increased over time (+US$4471 vs +US$4087, *p* < 0.001).

**Conclusions:** This program was associated with improved medication adherence and persistence, reduced multiple sclerosis-related hospitalization, and decreased multiple sclerosis-related medical costs. Unfortunately, the cost savings in the medical component did not offset the increased pharmacy expenditures during the 12-month follow-up period.

## Introduction

Multiple sclerosis (MS) is a chronic and progressive inflammatory disease of the central nervous system that affects approximately 211,000 to 400,000 people in the United States of America.^1–3^ It is characterized by many uncertainties. The cause is relatively unknown, the pathophysiological mechanisms are diverse, and the disease course is highly variable.^4,5^ In addition to the disease uncertainty and complexity, patients with MS experience a wide range of symptoms including fatigue, cognitive dysfunction, bowel and bladder dysfunction, weakness, spasticity, sexual dysfunction, and visual problems.^4,5^

Despite the availability of disease-modifying therapy (DMT) since 1993, the management of MS remains challenging. With no cure available, treatment and disease management focus on slowing progression and preventing relapse as well as controlling symptoms.^6,7^ Although the treatment efficacy has been established, adherence to pharmacotherapy remains one of challenges because of difficult side-effects. To meet this need and improve the management of MS, a specialty care management program has been introduced in one of the largest commercially insured populations in the USA since July of 2005. The intervention includes mailing medication and disease-specific patient education materials directly to the patients. Nurses make assessment calls at the beginning and follow-up assessments at months 3, 6, and 12, and every 12 months thereafter. They serve as a liaison to the pharmacy, a source of medical information, and a cheerleader to encourage adherence despite frequent difficult medication side-effects. The program also includes refill reminder calls. These calls enforce the importance of medication compliance, inquire about patient’s status and develop a relationship with the patient.

Many studies have reported the potential benefits of care management programs in other therapeutic areas such as diabetes, congestive heart failure, coronary artery disease, and asthma.^8–12^ However, relatively little published evidence about MS care management programs is available. Some earlier studies have shown the feasibility of programs for patient education, exercise, specialist nurse, depression disease management, and energy conservation among people with MS.^13–17^ Most of these studies were symptom specific and few were conducted on a wide scale. There is a need to understand the impact of this type of program and facilitate informed decision-making for review and implementation. This study aims to address this existing gap. The primary study hypothesis is that the specialty care management program is associated with medication adherence and persistence, risk of MS-related hospitalization, and MS-related cost of care. To our knowledge, this is the first study to evaluate the effectiveness of a specialty care management program on these clinical and economic outcomes among patients with MS.

## Methods

### Data source

This is a retrospective cohort study using administrative claims data from the HealthCore Integrated Research Database (HIRD^SM^), which includes 13 geographically dispersed US commercial health plans, providing coverage for approximately 24.2 million members. The administrative data set consists of integrated medical claims, pharmacy claims, and eligibility files. The study database was developed in compliance with Health Insurance Portability and Accountability Act of 1996 regulations (HIPAA).

### Patient selection

The study population included those members aged 18 or above with at least two medical claims with an *International Classification of Diseases, Ninth Revision, Clinical Modification* (ICD-9-CM) code for MS (ICD-9-CM: 340.xx) and at least one medical or prescription claim for MS medication between 1 January 2004 and 30 April 2008. MS medications of interest included interferon beta-1a (both intramuscular (IM) and subcutaneous (SC)), interferon beta-1b, glatiramer acetate, mitoxantrone, and natalizumab. For patients enrolled in the specialty care management program (participant group), the index date was defined as the start date of program participation. As other patients (non-participant group) did not have a program participation start date, an index date was randomly assigned through Monte Carlo simulation with regard to the participant patients on treatment history (length of time from the first observed MS treatment to program participation) to maximize the comparability of treatment history between participant and non-participant groups for subsequent evaluation. Non-participants included those who elected to obtain their medications from other sources, those who resided in geographical regions where the care management was not implemented at the time of this study, and/or those who participated in other care management programs which cannot be identified in the database. All patients were required to have ≥12 months of continuous health plan enrollment prior to and after index date.

### Study measures

Three outcomes were assessed: medication adherence and persistence, MS-related hospitalization, and total MS-related cost of care during the 12 months post-index period. Medication adherence was measured using medication possession ratio (MPR), defined as the ratio of the total days supply of medication dispensed during the period to the total number of days over the post-index 12-month period.^18,19^ Medication persistence referred to the duration of time from initiation to discontinuation of therapy,^18,19^ where discontinuation was defined as failing to obtain any MS medication within 60 days after the depletion of the previous days’ supply. MS-related hospitalization was measured through identification of inpatient hospitalizations from medical claims in which there were any claims containing an ICD-9-CM code for MS. Total MS-related cost of care reflected the total allowable amount reimbursed by the health plans, which consisted of both medical and pharmacy components. The medical component included cost incurred in inpatient, emergency room, and outpatient settings associated with an ICD-9-CM code for MS. The pharmacy component referred to the cost of MS medications filled or administered (interferon beta-1a, interferon beta-1b, glatiramer acetate, mitoxantrone, and natalizumab).

### Statistical analyses

All outcome measures were compared between the participant and non-participant groups. Statistical differences between groups were assessed using Wilcoxon rank-sum test for continuous variables and Pearson Chi-square test for categorical variables. An a priori two-tailed level of significance (alpha value) was set at the 0.05 level for all analyses.

Multivariate analyses were performed to evaluate the association between the intervention of the specialty care management program and each outcome while controlling for potential confounders. Generalized linear models were constructed using various response probability distributions and link functions, depending on the distribution of the outcomes and goodness of fit of the models. For instance, gamma distribution and log link function were used for total cost of care, while binomial distribution and logit link function were used for hospitalization. Covariates were chosen a priori for all models based on clinical relevance and baseline differences, which included age, gender, geographical region, plan type (i.e. health maintenance organization (HMO), preferred provider organization (PPO)), Deyo–Charlson co-morbidity index, co-morbid conditions (numbness, fatigue, abnormality of gait, and depression), the first MS medication on or after index date (i.e. interferon beta-1a, glatiramer acetate), length of treatment history (number of days between the first observed MS medication and index date), and baseline health care utilization (hospitalization and cost).

## Results

A total of 3993 patients with MS were identified. Of them, 78.3% (*n* = 3125) participated in the specialty care management program and 21.7% (*n* = 868) did not. Overall, the mean age was 46.3 ± 9.6 years and approximately three-quarters of the cohort (75.2%) were female. Interferon beta-1a IM was the most common MS medication used (37.3%), followed by glatiramer acetate (31.4%), interferon beta-1a SC (16.6%), interferon beta-1b (13.5%), natalizumab (0.8%), and mitoxantrone (0.5%). The participants and non-participants were comparable in terms of age, Deyo–Charlson co-morbidity index, and baseline co-morbid conditions (Table 1). Compared with non-participants, the participant patients had slightly more females, were covered by different health plans types (i.e. more HMO), had different geographic distribution, and had slightly higher proportion of beta-1a IM and glatiramer acetate use. Based on the time from first observed MS medication to index date, the participant group had a slightly longer treatment history than the non-participant group (mean 16.8 months vs 14.6 months, respectively, *p* < 0.001).

**Table 1.** Baseline patient characteristics (*n* = 3993)

Patient Characteristics	Participant	Non-participant	*p*-value
Number of patients, *n* (%)	3125 (78.3)	868 (21.7)	
Age, mean ± SD (median)	46.4 ± 9.3 (47.1)	45.9 ± 10.5 (46.1)	0.10
Female, %	76.4	71.0	0.001
Health plan region, %			< 0.001
East	15.6	24.9	
Central	36.5	20.9	
South	25.0	31.8	
West	22.9	22.5	
Health plan type, %			< 0.001
HMO	16.9	26.8	
POS	7.8	7.7	
PPO	69.1	52.9	
Others	6.21	12.6	
DCI score^a^, mean ± SD (median)	0.32 ± 0.85 (0)	0.34 ± 0.85 (0)	0.13
Co-morbid conditions, %			
Fatigue	16.8	17.3	0.74
Numbness	16.6	15.3	0.38
Depressive and mood disorders	41.7	44.1	0.21
Ataxia	3.0	1.8	0.06
Abnormality of gait	6.4	7.3	0.35
Fibromyalgia/myalgia and myositis	3.7	5.0	0.08
Urinary incontinence	3.6	2.5	0.13
Time from first observed MS treatment to index date (months), mean ± SD (median)	16.8 ± 10.1 (19.0)	14.6 ± 9.8 (16.4)	< 0.001
MS medication^b^, %			< 0.001
Interferon beta-1a IM	37.7	35.8	
Interferon beta-1a SC	16.7	16.0	
Interferon beta-1b	13.1	14.8	
Glatiramer acetate	31.8	29.8	
Natalizumab	0.5	1.8	
Mitoxantrone	0.2	1.7	

a Deyo–Charlson co-morbidity index.

b The first observed MS medication on or after index date.

During the 12-month period after the index date, medication adherence was significantly better in the participant group than the non-participant group (mean MPR: 0.86 vs 0.64, *p* < 0.001, Table 2). Compared with the 12-month period prior to index date, the MPR increased significantly more among the participants than the non-participants (mean +0.08 vs −0.03, *p* < 0.001). In multivariable regression analysis controlling for pre-index characteristics (including pre-index MPR), the participant group on average had 0.18 (95% confidence interval (CI): 0.16–0.19) higher MPR than the non-participant group. Similarly, the participants were more persistent on MS therapy (time from initiation to discontinuation of therapy) than the non-participants (mean 306.1 days vs 246.9 days, *p* < 0.001, Table 2). Over time, the average time from initiation to discontinuation of therapy of participant group increased by 29.4 days, while it decreased by 9.2 days in the non-participant group (*p* < 0.001). When pre-index characteristics were controlled for in multivariate analysis, the participants on average had a mean medication persistence of 50.6 (95% CI: 43.1–58.2) days longer than the non-participants.

**Table 2.** Medication adherence and persistence 12-month period prior to and after index date

Outcome Measures	Participant	Non-participant	*p*-value
MPR^a^			
Pre-index 12 months^b^, mean ± SD (median)	0.78 ± 0.28 (0.92)	0.68 ± 0.32 (0.82)	< 0.001
Post-index 12 months^c^, mean ± SD (median)	0.86 ± 0.20 (0.99)	0.64 ± 0.33 (0.74)	< 0.001
Change (post–pre)^d^, mean ± SD (median)	0.08 ± 0.31 (0.01)	−0.03 ± 0.30 (0)	< 0.001
Time from initiation to discontinuation of therapy^e^ (days)			
Pre-index 12 months^b^, mean ± SD (median)	275.0 ± 112.1 (336)	261.2 ± 125.3 (338)	0.76
Post-index 12 months^c^, mean ± SD (median)	306.1 ± 84.1 (343)	246.9 ± 129.6 (334)	< 0.001
Change (post–pre)^d^, mean ± SD (median)	29.4 ± 124.4 (12)	−9.2 ± 142.6 (0)	< 0.001

a Medication possession ratio.

b Among 88.9% (*n* = 2778) participants and 82.3% (*n* = 714) non-participants used one or more MS medication during this period.

c Among 99.1% (*n* = 3097) participants and 96.8% (*n* = 840) non-participants used one or more MS medication during this period.

d Among 88.4% (*n* = 2761) participants and 80.3% (*n* = 697) non-participants used one or more MS medication during both pre- and post- 12 months period.

e Discontinuation was defined as failing to obtain medication within 60 days after the depletion of the previous days supply.

**Figure 1.**
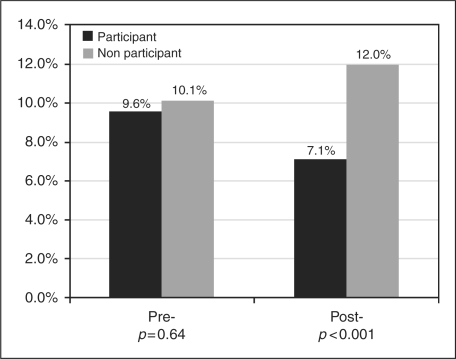
MS-related hospitalization 12-month period prior to and after index date.

During the 12-month period prior to the index date, participant and non-participant groups had comparable MS-related hospitalization rates (9.6% vs 10.1%, *p* = 0.64, Figure 1). However, the MS-related hospitalization rate became significantly lower for participants than that for non-participants during the 12-month period after index date (7.1% vs 12.0%, *p* < 0.001). Multivariable logistic regression analysis also showed the consistent trend that the participants were significantly less likely to have a MS-related hospitalization during the 12-month period after index date (adjusted odds ratio: 0.51, 95% CI: 0.39–0.67).

**Figure 2.**
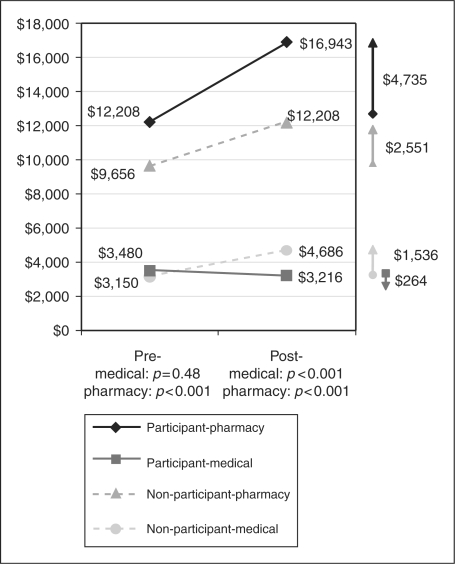
MS-related medical and pharmacy costs 12 month period prior to and after index date.

**Figure 3.**
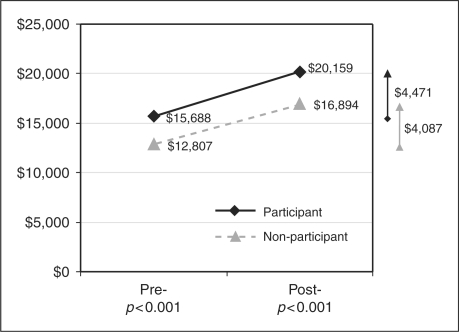
MS-related total cost of care 12-month period prior to and after index date.

Over 12 months, the average MS-related medical costs (non-pharmacy) decreased by US$264 among participants, while it increased US$1536 among non-participants (*p* < 0.001, Figure 2). On the other hand, the average MS-related pharmacy costs increased over time for both groups, with greater increases in the participant group (US$4735 vs US$2551, *p* < 0.001). Summing up the MS-related medical and pharmacy costs, the participant group had a larger increase in MS-related total cost of care from pre- to post-period than the non-participant group (US$4471 vs US$4087, *p* < 0.001, Figure 3). The same trend was found in multivariate analysis results. After adjusting for baseline characteristics, the participants had 21% (95% CI: 17%–26%) higher MS-related total cost of care than non-participants during the 12-month period after index date.

## Discussion

The MS specialty care management program had substantial impact on MS management in this large, commercially insured population. It was associated with improved medication adherence and persistence, reduced MS-related hospitalization, decreased MS-related medical costs, and increased MS-related total cost of care. These findings were encouraging, especially given the relatively short follow-up period of 1 year. Many times, the effects of disease management take years to become evident in patient outcomes, utilization of health care services, and economic outcomes.^9^

One year after enrollment of the program, the participants’ medication adherence and persistence were improved. Even though participants’ adherence appeared to be better at the baseline, it improved significantly more within a year (on average increased 29.2 days, converted from 0.08 MPR) while the adherence deteriorated among non-participants. This observation was further substantiated with similar findings in persistence on medication. Given equivalent persistence at the baseline, the participants’ persistence on MS medication improved during the 12-month period after index date, whereas the non-participants’ persistence worsened in the same period ( +29.4 days vs −9.2 days). Adherence has been crucial for obtaining the beneficial effects of treatment, as is true for other therapeutic areas.^20^ These results suggested that the program had a positive impact on patient adherence and persistence with MS medication, and might consequently obtain greater treatment benefits.

During the 12-month period after enrollment, the MS-related hospitalization rate for the participant group dropped from 9.6% to 7.1%, whereas it increased from 10.1% to 12.0% in the non-participant group. This considerable reduction in hospitalization risk among participants was encouraging, especially given that both groups had comparable MS-related hospitalization rates during the baseline period (9.6% vs 10.1%, *p* = 0.64). This reduction in MS-related hospitalizations represents a successful improvement in clinical benefits of the program, as these hospitalizations serve as an indicator of severe MS exacerbation and acute care service. The reduced risk also coincided with improved adherence and persistence among participants in the same period, which suggested that the improved clinical outcomes might be a result of better control in disease progression.

After 1 year of enrollment in the program, the participants decreased their average MS-related medical spending by US$264, as opposed to non-participants who increased it significantly by US$1536. This showed the potential cost savings of the program by avoiding unnecessary medical utilization including hospitalization, emergency department, and unscheduled office visits. On the other hand, the pharmacy expenditure increased in both groups over time, with greater magnitude among participants. The greater increase was expected in part because of improved medication adherence and persistence among participants. When summing up MS-related medical and pharmacy costs, however, the savings in medical utilization, unfortunately, did not offset the increased pharmacy spending for participants during the 12-month post-observation period. This could be largely explained by the fact that MS medications constituted about 72.3% (non-participants) to 84.0% (participants) of the MS-related total cost of care, which was in line with other studies showing that pharmacy costs accounted for the majority of the MS-related total cost.^21,22^ As a result, it is unlikely that a long-term cost saving will be seen in MS-related total cost for participants, unless there is a significant drop in medication costs in the future. Nonetheless, it is important to note that such a program is worthwhile even if it does not lead to overall cost reduction. In fact, it does not discount the clinical benefits observed in medication adherence and hospitalization in this study.

This study did not evaluate patient-reported outcomes. Thus, the effect of the program on other outcomes such as functional status and absenteeism was unclear. Other studies had shown the potential impact of care management program on quality of life, lost work/school days, and patient satisfaction in other therapeutic areas.^10,23–25^ One could speculate that the improved medication adherence and persistence, reduced risk of MS-hospitalization, and decreased MS-related medical spending observed in this study might have affected patients’ quality of life, productivity, and disease progress. With decreasing medical care utilization indicated by both reducing MS-related hospitalizations and medical spending, patients’ mobility level and functional status may have been better controlled and disease progress may have slowed down. As a result, we hypothesize that the long-term health of these MS patients would be more stable than if they had not participated in the program. The reduction of medical utilization also leads us to believe that the MS patients would have fewer MS-related absences from work and family-related responsibilities. However, further research will be needed to substantiate these hypotheses.

To validate the robustness of the study findings, we performed several sensitivity analyses with different methods. These methods included a) using all-cause hospitalization instead of MS-related hospitalization; b) using all-cause total cost instead of MS-related total cost; c) excluding patients over 65 years old; and d) excluding patients on mitoxantrone and natalizumab. All results of these sensitivity analyses were consistent with the primary results and did not alter the overall conclusions of this study.

### Limitations

The study findings are subject to several limitations. First, randomization of intervention (care management) was not performed due to the nature of observational study. Although the comparability between participants and non-participants in unobserved characteristics (such as MS types (i.e. relapsing–remitting, primary–progressive), disability level) cannot be assumed, the fact that over time change comparison still reflected the potential impact of the program increases the chance that the current results are robust. Second, misclassification or measurement error could occur in administrative claims, but it is unlikely that such error would be systematically different across cohorts. Third, the disenrollment information from the program was not available. The participant group might have included those who only partially participated or dropped out sometime after enrollment in the program, and hence the program effect observed in this study could be underestimated. Fourth, it could be argued that the results might not be generalizable to non-participants due to difference in unmeasured characteristics such as self-motivation and health-consciousness. The participants may already have been more motivated so that the management program was an add-on. Nonetheless, this does not refute the observation that the program affects participants as an aggregate, who accounted for 78.3% in this commercially insured population.

## Conclusions

To our knowledge, this study was the first to evaluate the impact of a MS specialty care management program on clinical and economic outcomes. Our findings suggested that this program was associated with improved medication adherence and persistence, reduced risk of MS-related hospitalization, and decreased MS-related medical costs (excluding pharmacy) over time. This represented an important metric of program success. It was also associated with higher MS-related total cost of care, which was primarily driven by high pharmacy costs. The cost saving in medical utilization, unfortunately, did not offset the increased expenditure in pharmacy during the 12-month period after the program intervention.
